# Polypeptide *N*-acetylgalactosaminyltransferase 6 expression in pancreatic cancer is an independent prognostic factor indicating better overall survival

**DOI:** 10.1038/bjc.2011.166

**Published:** 2011-05-17

**Authors:** Z Li, S Yamada, S Inenaga, T Imamura, Y Wu, K-Y Wang, S Shimajiri, R Nakano, H Izumi, K Kohno, Y Sasaguri

**Affiliations:** 1 Department of Pathology and Cell Biology, School of Medicine, University of Occupational and Environmental Health, 1-1 Iseigaoka, Yahatanishi-ku, Kitakyushu 807-8555, Japan; 2 Department of Pathology, Kyushu Kosei-Nenkin Hospital, Kitakyushu 806-8501, Japan; 3 Department of Surgery, Tobata Kyoritsu Hospital, Kitakyushu 804-0093, Japan; 4 Department of Molecular Biology, School of Medicine, University of Occupational and Environmental Health, Kitakyushu 807-8555, Japan; 5 Department of Medical Oncology, The First Hospital of China Medical University, Shenyang 110001, People's Republic of China

**Keywords:** pancreatic cancer, GalNAc-T6, GalNAc-T3, prognosis, metastasis

## Abstract

**Background:**

The family of polypeptide *N*-acetylgalactosaminyltransferases (GalNAc-Ts) is responsible for the altered glycosylation in cancer. The purpose of our study was to investigate the clinical significance of two isoforms, GalNAc-T6 and -T3, and their correlation with the prognosis of pancreatic cancer.

**Methods:**

Immunohistochemistry was used to analyse GalNAc-T6 and -T3 expressions in 70 clinicopathologically characterised pancreatic cancer cases.

**Results:**

Positive expressions of GalNAc-T6 and -T3 were immunohistochemically identified in 51% (36 of 70) and in 77% (54 of 70) of patients, respectively. A close relationship was noted between GalNAc-T6 positive expression and pathological well/moderate differentiated type (*P*=0.001), small tumour size (*P*=0.044), absence of vascular invasion (*P*=0.009), and low stage of the American Joint Committee on Cancer systems (*P*=0.043). The expression of GalNAc-T3 significantly correlated with good differentiation (*P*=0.001), but not with other clinicopathologic features. Furthermore, univariate and multivariate analyses revealed that GalNAc-T6 expression was an independent prognosis indicator for the disease, whereas GalNAc-T3 expression had no impact on clinical outcome, even though 33 of 36 GalNAc-T6-positive cases also had a positive expression of GalNAc-T3 (*P*=0.001, *r*=0.356).

**Conclusion:**

Both GalNAc-T6 and -T3 expressions correlated significantly with tumour differentiation, whereas only GalNAc-T6 expression predicted prognosis in pancreatic cancer.

Pancreatic cancer is responsible for ∼227 000 deaths per year in the world (Parkin *et al*, 2005). The 5-year overall survival rate for all patients diagnosed with pancreatic cancer is <4%. At present, surgical resection offers the only chance of cure, whereas long-term survival is rare, with the overall 5-year survival rates ranging from 10% to 25% (Cleary *et al*, 2004; Katz *et al*, 2008). Various clinicopathologic features, such as tumour size, and vascular involvement in resectable tumours, or initial patient performance status and the absence of distant metastasis in unresectable tumours, have been proposed as prognostic indicators, although the results remain inconsistent and inconclusive to date. The clinical importance of biological markers is also under evaluation. For example, most famously, initial serum levels of carbohydrate antigen (CA) 19-9, and more recently, Mucin-4, have been shown to be independent prognosis indicators of pancreatic cancer (Saitou *et al*, 2005; Berger *et al*, 2008). All these factors are useful guides in standardised clinical management and in establishing an individualised treatment plan for a patient.

Carbohydrate antigen 19-9 and mucins belong to cell-surface CAs, which comprise various categories of tumour markers and are essential in monitoring cell growth and the invasive and metastatic status of solid tumours. In mammals, the most common forms of CAs are the mucin-type O-glycans, which may constitute up to 80% of them (Bresalier, 1994; Brockhausen, 1999; Hollingsworth and Swanson, 2004; Nagata *et al*, 2007). The aberrant glycosylation of mucin-type O-glycans is thought to be associated with the functional alteration of cancer cells, including their antigenic and adhesive properties, as well as their potential for invasion and metastasis (Brockhausen, 2006). The enzymes responsible for glycosylation of CA – the glycosyltransferases and glycosidases – thus gain more and more attention (Ohtsubo and Marth, 2006). Here, we focus on the family of polypeptide *N*-acetylgalactosaminyltransferases (GalNAc-Ts) which catalyses the transfer of *N*-acetyl-*α*-D-galactosamine:polypeptide (GalNAc) from the sugar donor uridine diphosphate (UDP)-GalNAc to the serine and threonine residues of glycoproteins to synthesise the sugar chains which catalyse the initial step in the synthesis of mucin-type O-glycans (Marth, 1996).

Despite this seemingly simple function, the GalNAc-Ts form a big family which includes at least 15 distinct members and as many as 24 human isozymes. They are localised differentially within cellular compartments, display tissue-specific expression, and have different, although partly overlapping, kinetic properties and acceptor substrate specificities (Ten Hagen *et al*, 2003). Aberrant expression of the GalNAc-T isoforms results in aberrant O-glycosylation and participates in the progression of cancer (Hollingsworth and Swanson, 2004). According to the previous literature, GalNAc-T1 was considered to decrease ovarian cancer risk (Phelan *et al*, 2010), and the higher expression levels of GalNAc-T14 correlated with lower histological grade in breast invasive ductal carcinoma and tumour progression (Wu *et al*, 2010). The most extensively studied GalNAc-T was GalNAc-T3, which was proved by our group and others to be expressed in adenocarcinoma cell lines, but not in other carcinoma cell lines (Sutherlin *et al*, 1997; Nomoto *et al*, 1999). Recent reports showed that the expression of GalNAc-T3 was a useful indicator of prognosis in patients with colorectal, gastric, and lung cancer (Shibao *et al*, 2002; Onitsuka *et al*, 2003; Gu *et al*, 2004). GalNAc-T6 exhibited a high sequence similarity to GalNAc-T3 in the coding region and shared similar kinetic properties and acceptor substrate specificities which are distinct from the other GalNAc-Ts (Bennett *et al*, 1999). The expression of GalNAc-T6 has been reported in most ductal breast carcinomas showing a significant correlation with T1 stage (Berois *et al*, 2006), whereas in gastric carcinomas it is significantly associated with the presence of vascular invasion (VI) (Gomes *et al*, 2009). GalNAc-T6 thus could be considered as an intriguing marker of glycosylation modifications in cancer. To date, however, no study has assessed GalNAc-T6 expression and its correlation with GalNAc-T3 in pancreatic cancer.

In this study, we evaluated the expressions of GalNAc-T6 and -T3 in pancreatic cancer specimens and their correlation with the clinicopathologic features of the patients. The results indicated that GalNAc-T6 and -T3 were highly expressed in half of human pancreatic cancer cases, all of which were well to moderately differentiated type, while no or very weak staining was observed in normal pancreas ductal epithelium or poorly differentiated cancer. Furthermore, positive expression of GalNAc-T6, but not GalNAc-T3, is associated with good clinical prognosis of patients.

## Patients and methods

### Patient information, tumour specimens, and blood samples

The use of specimens from human subjects was approved by the Ethics Committee of the University of Occupational and Environmental Health (UOEH) Medical Center. Pathology reports were reviewed to identify patients who had undergone pancreaticoduodenectomy or distal pancreatectomy for pancreatic cancer between 1994 and June 2009 at the hospital of UOEH. Two patients who had suffered perioperative deaths, defined as death during the patient's initial hospitalisation or within 30 days of surgery, were excluded. A total of 70 patients with available follow-up data comprised the cohort of this retrospective study. Three pathologists examined all the resected specimens to confirm the histopathologic features. The tumour node metastasis system of the American Joint Committee on Cancer (AJCC) 6th edition was used for staging. Clinical information was gathered from the patients’ records, and the survival duration was set as the period from the date of surgery to death or the most recent clinic visit. Formalin-fixed, paraffin-embedded tissue blocks were obtained from our department of pathology. Serum CA19-9 and carcinoembryonic antigen (CEA) levels were measured at the time of final diagnosis. Normal human tissue was obtained from non-tumour portions of the surgically resected specimens.

### Preparation of antibody against GalNAc-Ts

Polyclonal antibody against GalNAc-T6 was raised by multiple immunisations of New Zealand white rabbits with synthetic peptides. The sequence of the synthetic peptide was GFYTPAELKPFWERPPQDP. The specificity of antibody was confirmed by western blotting and immunohistochemistry with peptide competition. Anti-GalNAc-T3 polyclonal antibody was generated in the same way as described previously (Nomoto *et al*, 1999).

### Immunohistochemistry of tissue samples

Immunohistochemical staining was performed by the antibody-linked dextran polymer method (EnVision; Dako Cytomation, Inc., Carpinteria, CA, USA). Deparaffinised and rehydrated 5 *μ*m sections were incubated in 3% H_2_O_2_ for 10 min to block endogenous peroxidase activity. The sections were then rinsed and incubated with rabbit polyclonal GalNAc-T6 or -T3 antibody for 30 min. The second antibody-peroxidase-linked polymers were then applied, and the sections were incubated with a solution consisting of 20 mg of 3.3′-diaminobenzidine tetrahydrochloride, 65 mg of sodium azide, and 20 ml of 30% H_2_O_2_ in 100 ml of Tris-HCl (50 mM, pH 7.6). After having been counterstained with Meyer's haematoxylin, the sections were observed under a light microscope. For immunohistochemistry of the neoplastic tissue, positive areas comprising <10% of the neoplasms were considered as negative staining. Positive areas that were equal to or >10% were defined as positive staining and were graded into three categories: weak, positive area of 11–30% moderate, 31–80% and strong, >80% positive area.

### Cell cultures

Human pancreatic invasive ductal carcinoma cell lines PANC1 (American Type Culture Collection) and PK1 (a kind gift from the Institute of Development, Aging and Cancer, Tohoku University, Japan) were maintained in a DMEM medium containing 10% fetal calf serum (GIBCO BRL, Rockville, MD, USA) at 37°C in an atmosphere of 95% air and 5% CO_2_.

### Immunofluorescence of pancreatic cancer cell lines PANC1 and PK1

The pancreatic cancer cell lines PANC1 and PK1 were cultured on coverslips, fixed with 95% acetone for 5 min, and allowed to air dry. The cells were then incubated with anti-GalNAc-T6 antibodies for 1 h at room temperature (RT), washed with PBS, and reacted with fluorescein isothiocyanate-conjugated goat anti-rabbit IgG for 1 h at RT. After having been washed with PBS, the specimens were observed under a Nikon ECLIPSE E600 inverted fluorescence microscope (Nikon, Tokyo, Japan).

### Cell fractionation and western blotting

The cells were washed and lysed with RIPA buffer. For nuclear and cytoplasmic fractions, the cells were scraped in 100 *μ*l ice-cold PBS and centrifuged at 500 **g** for 5 min. The cell pellet was resuspended in 100 *μ*l Buffer A (50 mM NaCl, 10 mM HEPES pH 8.0, 500 mM sucrose, 1 mM EDTA, 0.2% Triton X-100, freshly added protease inhibitors and 7 mM 2-mercaptoethanol), vortexed at high speed for 45 s and centrifuged at 2000 **g** for 2 min at 4°C. The resulting supernatant was used as a cytoplasmic extract. The pellet was resuspended in 100 *μ*l Buffer B (Buffer A with 25% glycerol and 0.1 mM EDTA) and nuclei were pelleted at 2000 **g** for 2 min at 4°C. The pellet was dissolved in 50 *μ*l Buffer B, incubated on ice for 30 min with intermittent high speed vortexing and spun down at 11 000 **g** for 15 min at 4°C. The supernatant was diluted to 100 *μ*l with PBS and used as a nuclear extract. Equal amounts of samples were analysed on an SDS–PAGE gel and then were transferred onto a nitrocellulose membrane. The membranes were incubated with the primary antibody and visualised with a secondary antibody coupled to horseradish peroxidase (Cell Signaling Technology, Beverly, MA, USA) and SuperSignal West Pico Chemiluminescent Substrate (Pierce, Rockford, IL, USA). The bands on the western blots were analysed densitometrically using Scion Image software (version 4.0.2; Scion Corp., Frederick, MD, USA).

### Statistical analysis

Spearman’s correlation test was used to assess the relationships between variables. Survival curves were plotted by the Kaplan–Meier method and compared with the log-rank test. Hazard ratios and 95% confidence intervals (95% CIs) were estimated using univariate or multivariate Cox proportional-hazard models. All statistical tests were two-tailed, with *P*<0.05 considered significant. SPSS statistical software (SPSS for Windows, version 16.0.0 (SPSS, Chicago, IL, USA)) was used for the above statistical analyses.

## Results

### Patient characteristics

As shown in Table 1, the cohort included 70 patients (40 men and 30 women) with clinicopathologic features representative of pancreatic cancer. The average age at surgery was 67 years. The median tumour size was 3.5 cm with a range from 1.0 to 7.4 cm. More than half of the patients (62.9%) had lymph node metastasis at diagnosis, and most of the tumours were graded as well to moderately differentiated type. Based on the AJCC criteria, the majority of the patients had stage II disease. Follow-up was available for all 70 patients, ranging from 3.1 to 70.0 months, with a median of 14.1 months. The median overall postoperative survival duration was 15.9 months, with 1- and 5-year actuarial survival rates of 58% and 6%, respectively. The records of serum CA19-9 and CEA levels were available for 59 and 60 cases in the cohort, respectively. Supplementary Table 1 displays each patient's information in detail.

### Expression of GalNAc-Ts in normal tissue and pancreatic cancer specimens

**Figure 1:**
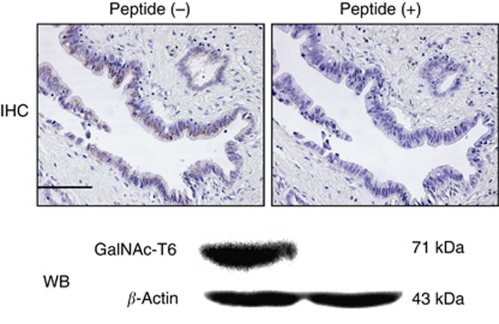
Immunohistochemical and western blotting analyses of GalNAc-T6 expressions in well-differentiated pancreatic cancer sample ( × 200) and PANC1 cell line after incubation of an anti-GalNAc-T6 polyclonal antibody with or without synthesised peptides of GalNAc-T6. IHC, immunohistochemistry; WB, western blotting. Bar, 100 *μ*m. GalNAc-T6 expression is completely abolished with peptide.

**Figure 2:**
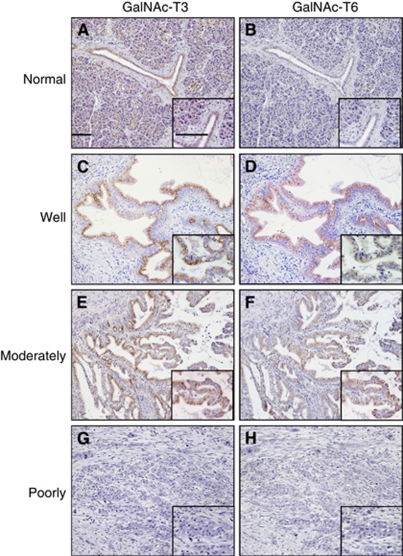
Immunohistochemical analysis of GalNAc-T3 (1 : 3000 dilution) and GalNAc-T6 (1 : 1000 dilution) in human pancreatic cancers and normal ductal specimens ( × 100; inset, × 400). Bar, 100 *μ*m. Normal pancreatic duct was positive for GalNAc-T3 (**A**), but negative for GalNAc-T6 (**B**). Well and moderately differentiated pancreatic cancers stain positively with GalNAc-T3 (**C** and **E**) and GalNAc-T6 (**D** and **F**), respectively. However, poorly differentiated cancer shows negative staining with both GalNAc-T3 (**G**) and GalNAc-T6 (**H**).

The specificity of the GalNAc-T6 polyclonal antibody was tested by immunohistochemistry and western blotting. After incubation of this antibody with the excess of synthesised peptides of GalNAc-T6, the positive immunostaining was completely abolished (Figure 1). Immunohistochemically, GalNAc-T3 and -T6 showed only cytoplasmic expressions (Figure 2). The expression of GalNAc-T3 was present in most of the normal ductal epithelium samples (Figure 2A), whereas GalNAc-T6 expression was rare or very weakly detectable (Figure 2B). The expression of GalNAc-T3 was present in 54 of 70 (77%) pancreatic adenocarcinoma specimens: 38%, weak; 23%, moderate; and 16%, strong, respectively. The expression of GalNAc-T6 was present in 36 of 70 (51%) specimens: 34%, weak; 13%, moderate; and 4%, strong, respectively. When GalNAc-Ts expression was dichotomised into groups of either positive (weak to strong staining) or negative, the GalNAc-T3/-T6 immunoprofile was 19%, (−)/(−); 30%, (+)/(−); 4%, (−)/(+); and 47%, (+)/(+), respectively.

### Association of GalNAc-Ts expression with clinicopathologic variables

To identify the association of GalNAc-Ts expression (GalNAc-Ts negative *vs* positive) with the clinical and pathological variables of the cohort, the variables were dichotomised as shown in Table 1. There were no significant differences between the patients with GalNAc-T3 negative and positive tumour expressions regarding age, gender, tumour location, size, lymph node involvement, disease stage, margin status, lymphatic invasion, VI, perineural invasion or serum CA19-9 or CEA level (*P*>0.05), whereas GalNAc-T3 staining status significantly affected tumour differentiation (*P*=0.001; Figures 2C, E, and G). The rate of well to moderately differentiated tumours in the GalNAc-T3-negative group was 10 out of 16 (62.5%), but 51 out of 54 (94.4%) in the GalNAc-T3-positive group. These data were consistent with the results of a previous study using the same antibody as ours (Yamamoto *et al*, 2004). Positive GalNAc-T6 staining also correlated significantly with well to moderately differentiated type (*P*=0.001; Figures 2D, F, and H). Moreover, it was associated with early disease stage and absence of VI (*P*=0.043 and 0.009, respectively) in all the tumours and with decreasing tumour size in the resectable tumours (*P*=0.044). Thirty out of thirty-six cases (83.3%) in the GalNAc-T6-positive group were classified into stage I/II of the AJCC TNM classification, compared with 21 out of 34 (61.8%) in the GalNAc-T6-negative group.

**Figure 3:**
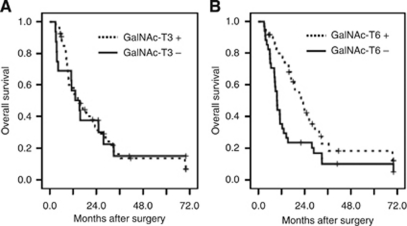
Kaplan–Meier curves of overall survival durations in patients with pancreatic cancer after surgery according to GalNAc-T3 (**A**) and GalNAc-T6 (**B**). Pancreatic cancer patients have similar survival regardless of GalNAc-T3 expression level (*P*=0.956), however, patients with positive GalNAc-T6 expression have significantly longer survivals than those with negative GalNAc-T6 expression (*P*=0.014).

The 5-year overall survival rate of the cohort was 6%. In a Kaplan–Meier analysis (Figure 3), GalNAc-T3 expression showed no prognostic significance in overall survival. In contrast, patients with GalNAc-T6-positive expression had significantly longer overall postoperative survival (median, 21.8 months) compared with those who had GalNAc-T6-negative expression (median, 9.3 months; *P*=0.014). For the patients with GalNAc-T6-positive tumours, an increasing degree of GalNAc-T6 expression was not associated with better clinicopathologic features or longer survival (data not shown).

We also evaluated survival rates for GalNAc-T6 status year by year (Supplementary Table 2). In patients with GalNAc-T6-positive expression, the 1- and 2-year survival rates were 80.6% and 41.7%, respectively. However, for patients without GalNAc-T6 expression, the survival rates were much less; the rates were 35.3% and 31.4% (*P*=0.0003 and 0.006, respectively). Positive expression of GalNAc-T6 also showed a significant but modest survival advantage compared with the 3-, 4-, and 5-year survival rates (*P*=0.028, 0.048, and 0.035, respectively).

### GalNAc-T6 represents an independent prognostic indicator in pancreatic cancer

To assess whether GalNAc-T6 expression was an independent predictor of overall postoperative survival, a Cox proportional-hazards model was created in a forward manner including only the covariates that had a statistically significant correlation (inclusion threshold, *P*<0.05) with overall survival (Table 2). Univariate analysis demonstrated that poor tumour differentiation, advanced stage and GalNAc-T6-negative status were significant predictors of poorer survival (*P*=0.003, <0.001, and 0.014, respectively). Margin status had no significant impact on patient survival in our relatively small cohort (*P*=0.124).

Furthermore, multivariate analysis showed that, after correction for confounding variables, GalNAc-T6 expression remained an independent prognostic indicator of overall survival (*P*=0.024).

### Analysis of GalNAc-T6 expression in human pancreatic cancer cell lines

**Figure 4:**
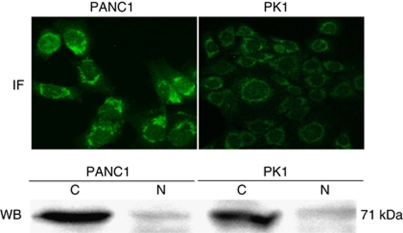
Immunofluorescent and western blotting analyses of GalNAc-T6 in pancreatic cancer cell lines PANC1 and PK1. IF, immunofluorescence. WB, western blotting. The cytoplasmic expression levels of GalNAc-T6 are significantly higher in primary cancer cell line PANC1 than those in metastatic cancer cell line PK1 with both methods.

The immunofluorescence staining of the pancreatic adenocarcinoma cell lines (PANC1 and PK1) showed a marked expression of GalNAc-T6 in the cytoplasm in a perinuclear manner (Figure 4). Western blotting analysis showed GalNAc-T6 expression in the cytoplasm, whereas very weak nuclear expression was detectable in both cell lines (Figure 4). Additionally, the expression levels of GlaNAc-T6 in PANC1 were significantly higher than those in the PK1 cell line by both methods.

## Discussion

Prior studies revealed that GalNAc-T6 expression was detected in a few kinds of malignancies, and breast cancer was the most studied type in this field. Interestingly, one group showed that GalNAc-T6 was expressed in most ductal carcinoma *in situ* and was significantly associated with the T1 stage rather than higher stage tumours (Berois *et al*, 2006). Other studies showed that GalNac-T6 mRNA positively correlated with bone marrow metastasis (Freire *et al*, 2006), and knockdown of GalNAc-T6 suppressed the growth of breast cancer cells (Park *et al*, 2010). These conflicting results are probably due to the heterogeneity of breast cancer, which can be supported by the fact that distinctive chemotherapy drugs showed good efficacy on it. By contrast, pancreatic cancer has been supposed to be a relatively homogeneous cancer with a low frequency of either molecular or histological variants (Niederhuber *et al*, 1995) and would be a better model to demonstrate the function of GalNAc-T6 in malignancies. Thus, we examined the clinical relevance of GalNAc-T6 expression in pancreatic cancer. Expression of GalNAc-T6 was observed in half of pancreatic cancer specimens, associated closely with well to moderately differentiated type (*P*<0.001), VI (*P*=0.009), early AJCC stage (*P*=0.043), as well as small tumour size in resectable cases (*P*=0.044). In both univariate and multivariate analyses, a positive GalNAc-T6 status predicted improved overall survival. To our knowledge, this is the first study to reveal that GalNAc-T6 was an independent prognostic indicator in patients with pancreatic cancer.

Further analysis of the correlation between GalNAc-T6-positive expression and better 1-year or 2-year overall survival (*P*=0.0003 and 0.006, respectively; Supplementary Table 2) indicated that positive GalNAc-T6 expression significantly predicted a survival advantage, especially in the early phase after resection, very similar to postoperative serum CA19-9 (Berger *et al*, 2008). Thus, negative GalNAc-T6 status recommended more aggressive treatment. Because GalNAc-Ts are in external secretions such as colostrum, based on the previous literature (Hagen *et al*, 1993; Bennett *et al*, 1996; Kitazume *et al*, 2001), it is very likely that GalNAc-T6 could be secreted to body fluids too. Therefore, GalNAc-T6 is considered as a useful candidate for surveillance because it is well known that >80% postoperative relapse (local or distant) occurs within the first 2 years (Stathis and Moore, 2010).

Additionally, the hypothesis that GalNAc-T6 could be a good indicator of local aggression and metastasis in pancreatic cancer is supported by our result that GalNAc-T6 was negatively associated with VI (*P*=0.009, *r*=−0.181). Not only that, but the primary pancreatic cancer cell line (PANC1) demonstrated, by western blotting and immunocytochemistry methods, a significantly higher expression of GalNAc-T6 (Figure 4) than that of PK1 derived from liver metastasis (Kobari *et al*, 1984; Motomura *et al*, 2000), which was considered as a more aggressive subtype. These results were consistent with the data of Bennett *et al* (1999) in which northern blotting analysis showed that GalNAc-T6 mRNA could only be detected in one primary cancer cell line (miaPaca2), but not in other metastatic cancer cell lines such as ASPC1 and COLO357. Very similarly, our preliminary data showed a relatively higher expression of GalNAc-T6 in primary colon cancer specimens than that in asynchronous liver metastatic specimens (data not shown).

The molecular mechanism to explain this tight relationship between GalNAc-T6 status and local invasion or metastasis remains to be elucidated. However, recent studies focusing on the modulation of GalNAc-Ts to cell adhesion function, as well as the degradation of connective tissue, provide some useful clues. Several GalNAc-Ts were shown to affect leukocyte adhesion through modulating E- and P-selectin counter receptors (Lowe, 2003; Tenno *et al*, 2007); polypeptide GalNAc-T3, in Drosophila, was found to regulate integrins and to promote cell adhesion (Zhang *et al*, 2008, 2010); and GalNAc-T3 was found to modulate the activities of metalloproteinases (Chefetz *et al*, 2009). GalNAc-T6 was also found to regulate the cell adhesion molecular E-cadherin and *β*-catenin in breast cancer (Park *et al*, 2010). Therefore, it is reasonable to hypothesise that GalNAc-T6 can suppress invasion and metastasis by impacting cell–cell adhesion and cell–stromal interaction in pancreatic cancer, and we attribute this to the early survival advantage of GalNAc-T6 expression mentioned above.

Interestingly, we found that positive GalNAc-T6 expression was closely associated with GalNAc-T3 positivity (*P*=0.002, *r*=0.356) in our cohort. We used the anti-human polyclonal GalNAc-T6 antibody raised against a completely distinctive synthetic peptide (GFYTPAELKPFWERPPQDP) from GYYTAAELKPVLDRPPQDS of GalNAc-T3 for both immunohistochemistry and western blotting (Nomoto *et al*, 1999). The results revealed GalNAc-T6- or GalNAc-T3-specific staining in clinical specimens (Figurse 2A and B) and a distinct specific band of GalNAc-T6 (Figure 4) or GalNAc-T3 (data not shown) detected in the cytoplasmic fraction of pancreatic cancer cell lines, respectively. Additionally, the preliminary immunohistochemical examinations of different kinds of adenocarcinomas demonstrated ubiquitous expression of GalNAc-T3 in most specimens of lung, breast, gastric, and colon cancers, whereas GalNAc-T6 was expressed in only a few cases of them. All these confirmed the specificity of our GalNAc-T3 and -T6 antibodies.

Indeed, among all the isoforms, GalNAc-T6 and -T3 formed a subfamily with high sequence similarity in the coding region and shared similar kinetic properties and acceptor substrate specificities which are distinct from the other GalNAc-Ts (Bennett *et al*, 1999). It has been reported that there are at least two kinds of interaction – competition or complement – among the isoforms of GalNAc-Ts, as proven by experiments *in vitro* (Brockhausen *et al*, 1990; Bennett *et al*, 1998). Thus, the interaction of GalNAc-T6 and -T3 is a prospective topic awaiting illumination. At first, we hypothesised that the survival duration might be different between the double negative group and either positive group if the relationship between GalNAc-T3 and -T6 were complementary. On the other hand, if GalNAc-T3 and -T6 competed with each other, there might be some differences in the survival duration between the GalNAc-T3 positive-only group and the GalNAc-T6 positive-only group. Hence, the cohort was divided into two such manners (double negative *vs* either positive, and GalNAc-T3 positive-only *vs* GalNAc-T6 positive-only) and the Kaplan–Meier method was used to verify them. Unexpectedly, the overall survival of patients showed no significant difference (*P*=0.618 and 0.451, respectively) in either classification approach, suggesting that there was no apparent competitive or complementary correlation between GalNAc-T3 and -T6. Based on our analysis with a small cohort, GalNAc-T3 and -T6 expressed together, but might function separately, which was very interesting and needs further confirmation in larger cohorts later.

In summary, the present findings demonstrate that the positive expression of GalNAc-T6 in pancreatic cancer has a significantly close relationship with the well to moderately histopathologic phenotype, absence of VI, and high incidence of early stage of the AJCC system, namely well-differentiated and non-invasive characteristics. Moreover, the outcome of the patients who had tumours with positive GalNAc-T6 expression is significantly better than that with negative GalNAc-T6 expression, especially in the early period after surgery. On the other hand, most GalNAc-T6-positive cases expressed GalNAc-T3 as well, whereas they influenced overall survival separately. Based on these features, we can, for the first time, implicate that GalNAc-T6 is an independent novel and useful marker of prognosis in patients with pancreatic cancer, but GalNAc-T3 is not. Compared with more conventional markers like CA19-9, CEA, or mucins, GalNAc-T6 is suggested to have a potential but special advantage to predict not only the prognosis of patients, but local aggression and metastasis too. It is of clinical value to identify the danger of local invasion and metastasis, which are the primary causes of the poor outcome for this cancer. By contrast, we admit the disadvantage of this molecular marker. Its negative expression in undifferentiated cancer makes it difficult to be used as a diagnostic marker in pancreatic cancer. However, it might be a candidate for combination with the conventional markers mentioned above.

Actually, since this cohort was not big, margin status did not predict the ultimate outcomes in the univariate analysis. It is expected that the status and the prognostic value of GalNAc-T6 will be evaluated in a larger cohort. On the other hand, it is postulated that GalNAc-T6 had a key role in various types of cancer, thus evaluation of its prognostic value in other cancer types would be another important and intriguing topic. Furthermore, it is worth doing more molecular experiments on the potential invasive and metastatic suppressor function of GalNAc-T6 in pancreatic cancer. Finally, we plan to perform a high-throughput assay to evaluate the functions of other GalNAc-Ts in pancreatic cancers. It would be very interesting to further study the relationships between GalNAc-Ts and mucins.

## Figures and Tables

**Table 1:** Correlation between GalNAc-T3 or -T6 expression and clinicopathologic variables

	**Total**	**GalNAc-T3 positive, *n* (%)**		**GalNAc-T6 positive *n* (%)**	
**Variables**	***n* (%)**	**Positive, *n*=54**	***P***	**Positive, *n*=36**	***P***
*Age, years*			0.331		0.934
⩽60	17 (24.3)	14 (82.4)		7 (41.2)	
>60	53 (75.7)	40 (75.5)		29 (54.7)	
					
*Sex*			0.751		0.842
Male	40 (57.1)	32 (80.0)		22 (55.0)	
Female	30 (42.9)	22 (73.3)		14 (46.7)	
					
*Tumour differentiation*			0.001		0.001
Well, moderately	61 (87.1)	51 (83.6)		36 (59.0)	
Poorly	9 (12.9)	3 (33.3)		0 (0.0)	
					
*Tumour size, pathologic* a			0.751		0.044
⩽3.5 cm	30 (58.8)	24 (80.0)		21 (70.0)	
>3.5 cm	21 (41.2)	16 (57.1)		9 (42.9)	
					
*N classification*			0.085		0.760
Negative	26 (37.1)	23 (88.5)		14 (53.8)	
Positive	44 (62.9)	31 (70.5)		22 (50.0)	
					
*Stage (AJCC)*			0.679		0.043
I–II	51 (72.9)	40 (78.4)		30 (58.8)	
III–IV	19 (27.1)	14 (73.7)		6 (31.6)	
					
*Margin status*			0.607		0.161
Negative	39 (55.7)	31 (79.5)		23 (59.0)	
Positive	31 (44.3)	23 (74.2)		13 (41.9)	
					
*Lymphatic invasion*			0.269		0.954
Absent	4 (5.7)	4 (100.0)		2 (50.0)	
Present	66 (94.3)	50 (75.8)		34 (51.5)	
					
*Vascular invasion*			0.173		0.009
Absent	18 (25.7)	16 (88.9)		14 (77.8)	
Present	52 (74.3)	38 (73.1)		22 (42.3)	
					
*Perineural invasion*			0.676		0.066
Absent	3 (4.3)	2 (66.7)		0 (0.0)	
Present	67 (95.7)	52 (77.6)		36 (100.0)	
					
*GalNAc-T3 expression*			—		0.002
Negative	16 (22.9)	—		3 (18.6)	
Positive	44 (77.1)	—		33 (75.0)	
					
*GalNAc-T6 expression*			0.002		—
Negative	34 (48.6)	21 (61.8)		—	
Positive	36 (51.4)	33 (91.7)		—	

Abbreviation: AJCC=American Joint Committee on Cancer.

a On 51 resectable tumours.

**Table 2:** Univariate and multivariate analyses of survival in 70 patients with pancreatic cancer according to clinicopathologic variables and GalNAc-T3 or -T6 expression

	**Univariate**			**Multivariate**		
**Risk factors**	**Hazard**	**95% CI**	***P***	**Hazard**	**95% CI**	***P***
GalNAc-T3 expression	0.96	0.51–1.78	0.892			
GalNAc-T6 expression	0.52	0.30–0.88	0.014	0.53	0.31–0.92	0.024
Poor differentiation	3.22	1.48–7.00	0.003			
Tumour in head of pancreas	0.91	0.53–1.58	0.743			
Increasing tumour size	1.86	0.87–3.95	0.109			
Presence of LN metastasis	1.46	0.85–2.53	0.175			
Advanced stage (III/IV)	3.97	2.10–7.50	<0.001	3.65	1.86–7.16	<0.001
Positive tumour margin	1.49	0.89–2.51	0.132			
Presence of LI	1.62	0.90–2.88	0.103			
Presence of VI	1.27	0.73–2.20	0.400			
Presence of PNI	1.25	0.69–2.26	0.463			
Highly serum CA19-9 level	1.60	0.86–2.99	0.137			
Highly serum CEA level	1.44	0.71–2.93	0.318			

Abbreviations: CI=confidence interval; LN=lymph node; LI=lymphatic invasion; VI=vascular invasion; PNI=perineural invasion; CA19-9=carbohydrate antigen 19-9; CEA=carcinoembryonic antigen.
