# Supplementary material for: Polypeptide N-acetylgalactosaminyltransferase 6 expression in pancreatic cancer is an independent prognostic factor indicating better overall survival
Source: Br J Cancer. 2011 May 17;104(12):1882–9. doi: 10.1038/bjc.2011.166 (PMC3111199; doi:10.1038/bjc.2011.166)
Supplement: Supplementary Table 1 [file bjc2011166x1.doc]

**Supplementary Table 1.** The relationship between GalNAc-T3 or -T6 expressions and each patient’s characteristics

| **No.** | **Status** | **Duration(month)** | **-T3** | **-T6** | **Age** | **Gender** | **Location** | **Grade** | **Size(cm)** | **Tstage** | **Nstage** | **AJCC stage** | **Margin** |
| --- | --- | --- | --- | --- | --- | --- | --- | --- | --- | --- | --- | --- | --- |
| **1** | dead | 3.2 | - | - | 80 | female | head | poor | 5.5 | 4 | + | 3 | - |
| **2** | dead | 16.9 | + | + | 64 | female | body/tail | well | 3.5 | 2 | - | 1 | - |
| **3** | dead | 28.7 | + | - | 79 | female | body/tail | moderate | 4.4 | 3 | - | 2 | - |
| **4** | dead | 29.3 | + | + | 67 | male | body/tail | moderate | 1.0 | 3 | - | 2 | + |
| **5** | dead | 32.8 | + | + | 48 | male | head | well | 2.0 | 3 | + | 2 | + |
| **6** | dead | 8.4 | + | - | 71 | female | head | moderate | 3.0 | 2 | - | 1 | + |
| **7** | dead | 15.4 | - | - | 56 | female | head | poor | 5.5 | 4 | + | 4 | + |
| **8** | dead | 8.7 | + | - | 69 | female | head | poor | 2.0 | 3 | - | 2 | - |
| **9** | dead | 6.1 | + | - | 63 | female | head | well | 3.0 | 4 | + | 3 | - |
| **10** | dead | 11.1 | - | - | 72 | male | body/tail | poor | 3.0 | 3 | + | 4 | + |
| **11** | dead | 31.5 | + | + | 62 | male | body/tail | well | 3.0 | 3 | + | 2 | - |
| **12** | alive | 41.6 | + | + | 71 | male | body/tail | well | 2.5 | 2 | - | 1 | - |
| **13** | dead | 23.9 | + | + | 67 | male | head | well | 2.5 | 3 | + | 2 | - |
| **14** | dead | 6.6 | + | - | 73 | male | head | moderate | 3.5 | 4 | - | 3 | + |
| **15** | alive | 15.9 | + | + | 56 | male | head | moderate | 2.5 | 3 | + | 2 | - |
| **16** | dead | 6.2 | + | - | 71 | female | body/tail | poor | 3.0 | 4 | + | 3 | - |
| **17** | dead | 9.0 | + | - | 54 | male | head | well | 2.5 | 4 | - | 3 | - |
| **18** | alive | 70.0 | + | + | 75 | female | head | moderate | 5.5 | 3 | - | 2 | - |
| **19** | dead | 33.0 | + | - | 61 | female | body/tail | well | 5.0 | 3 | + | 2 | + |
| **20** | dead | 70.0 | + | - | 62 | male | head | moderate | 2.5 | 3 | - | 2 | + |
| **21** | dead | 21.3 | + | + | 70 | male | head | well | 3.0 | 3 | - | 2 | - |
| **22** | dead | 9.4 | + | + | 63 | male | head | moderate | 2.5 | 3 | + | 2 | - |
| **23** | dead | 32.9 | - | - | 68 | female | head | moderate | 4.5 | 3 | + | 2 | + |
| **24** | dead | 25.1 | - | + | 69 | female | head | moderate | 2.0 | 1 | + | 2 | - |
| **25** | dead | 9.7 | + | - | 71 | male | head | moderate | 5.0 | 2 | - | 1 | + |
| **26** | dead | 18.7 | + | + | 71 | female | head | moderate | 3.0 | 3 | - | 2 | + |
| **27** | dead | 22.2 | + | + | 69 | male | head | well | 3.5 | 3 | + | 2 | - |
| **28** | dead | 14.5 | + | - | 80 | female | head | moderate | 4.0 | 1 | + | 2 | + |
| **29** | dead | 3.6 | - | - | 75 | male | body/tail | moderate | 4.4 | 2 | + | 2 | - |
| **30** | dead | 11.2 | + | - | 59 | male | head | moderate | 4.5 | 3 | + | 2 | + |
| **No.** | **Status** | **Duration(month)** | **-T3** | **-T6** | **Age** | **Gender** | **Location** | **Grade** | **Size(cm)** | **Tstage** | **Nstage** | **AJCC stage** | **Margin** |
| **31** | alive | 70.0 | + | + | 56 | female | head | well | 1.0 | 1 | + | 2 | - |
| **32** | dead | 9.8 | + | - | 54 | male | head | moderate | 6.5 | 4 | + | 3 | + |
| **33** | dead | 3.0 | - | - | 64 | male | body/tail | poor | 4.0 | 3 | + | 2 | - |
| **34** | dead | 5.3 | + | + | 78 | female | head | moderate | 4.0 | 3 | + | 2 | - |
| **35** | dead | 8.6 | + | - | 60 | male | body/tail | well | 4.5 | 3 | - | 2 | + |
| **36** | dead | 12.6 | + | + | 67 | female | head | moderate | 3.0 | 3 | + | 2 | - |
| **37** | dead | 36.5 | + | + | 79 | male | body/tail | moderate | 3.5 | 1 | - | 1 | - |
| **38** | dead | 9.4 | + | + | 83 | female | head | moderate | 4.5 | 4 | + | 3 | - |
| **39** | dead | 19.0 | + | + | 60 | female | body/tail | well | 5.0 | 3 | - | 2 | + |
| **40** | dead | 3.8 | + | + | 77 | female | body/tail | moderate | 4.0 | 3 | + | 2 | + |
| **41** | dead | 13.7 | + | + | 49 | male | head | well | 4.5 | 4 | + | 3 | - |
| **42** | dead | 5.1 | + | - | 44 | male | head | moderate | 7.0 | 4 | - | 3 | - |
| **43** | dead | 8.6 | + | + | 66 | male | head | well | 7.5 | 4 | + | 3 | + |
| **44** | dead | 3.7 | - | - | 67 | female | head | poor | 3.5 | 3 | + | 2 | + |
| **45** | dead | 8.3 | + | - | 40 | male | head | moderate | 5.0 | 3 | + | 2 | - |
| **46** | dead | 3.1 | + | + | 66 | male | head | moderate | 5.0 | 3 | - | 4 | + |
| **47** | dead | 12.4 | + | - | 70 | female | head | moderate | 4.0 | 4 | + | 3 | + |
| **48** | dead | 27.7 | - | - | 66 | male | head | moderate | 3.0 | 2 | + | 2 | + |
| **49** | alive | 70.0 | - | - | 65 | male | head | moderate | 3.0 | 3 | + | 2 | - |
| **50** | dead | 6.0 | + | - | 52 | female | body/tail | moderate | 5.0 | 4 | + | 3 | + |
| **51** | dead | 7.6 | + | + | 71 | male | body/tail | moderate | 6.5 | 4 | + | 3 | + |
| **52** | dead | 13.2 | - | - | 54 | male | body/tail | well | 1.0 | 4 | + | 3 | + |
| **53** | dead | 11.3 | - | - | 53 | female | head | moderate | 3.5 | 3 | - | 2 | + |
| **54** | dead | 15.8 | + | + | 68 | female | head | moderate | 3.2 | 3 | + | 2 | - |
| **55** | dead | 69.5 | + | + | 75 | female | head | well | 1.5 | 3 | + | 2 | + |
| **56** | dead | 23.3 | + | + | 77 | male | body/tail | moderate | 4.5 | 3 | - | 2 | + |
| **57** | lost | 28.0 | + | + | 61 | male | head | moderate | 4.0 | 3 | - | 2 | - |
| **58** | alive | 40.9 | - | - | 67 | female | body/tail | moderate | 2.0 | 2 | - | 1 | - |
| **59** | dead | 26.3 | + | + | 64 | male | head | moderate | 1.8 | 3 | + | 2 | - |
| **60** | alive | 32.5 | + | + | 59 | female | body/tail | well | 2.7 | 3 | + | 2 | - |
| **61** | lost | 5.5 | + | + | 49 | male | head | moderate | 3.0 | 3 | + | 2 | + |
| **No.** | **Status** | **Duration(month)** | **-T3** | **-T6** | **Age** | **Gender** | **Location** | **Grade** | **Size(cm)** | **Tstage** | **Nstage** | **AJCC stage** | **Margin** |
| **62** | dead | 17.0 | + | + | 70 | male | head | moderate | 3.0 | 3 | - | 2 | - |
| **63** | dead | 9.7 | + | - | 67 | male | head | well | 2.0 | 3 | + | 2 | - |
| **64** | dead | 9.9 | + | - | 73 | female | body/tail | moderate | 6.2 | 4 | + | 3 | - |
| **65** | alive | 25.0 | - | + | 77 | female | head | moderate | 3.0 | 2 | + | 3 | - |
| **66** | alive | 22.1 | + | - | 76 | male | body/tail | poor | 4.0 | 2 | - | 1 | - |
| **67** | dead | 15.7 | - | + | 63 | male | body/tail | moderate | 4.0 | 3 | - | 2 | + |
| **68** | dead | 4.3 | - | - | 62 | male | body/tail | poor | 6.0 | 3 | + | 2 | - |
| **69** | lost | 6.0 | + | + | 68 | male | head | well | 5.0 | 3 | + | 2 | - |
| **70** | alive | 18.0 | + | + | 76 | male | body/tail | well | 5.0 | 3 | - | 2 | + |
| -T3: GalNAc-T3; -T6: GalNAc-T6 | | | | | | | | | | | | | |
