# Supplementary material for: Polypeptide N-acetylgalactosaminyltransferase 6 expression in pancreatic cancer is an independent prognostic factor indicating better overall survival
Source: Br J Cancer. 2011 May 17;104(12):1882–9. doi: 10.1038/bjc.2011.166 (PMC3111199; doi:10.1038/bjc.2011.166)
Supplement: Supplementary Table 2 [file bjc2011166x2.doc]

Supplementary Table 2. The survival rates by GalNAc-T6 status

| Survival rate | -T6 Positive (%) | -T6 Negative (%) | P |
| --- | --- | --- | --- |
| 1-year | 80.6 | 35.3 | 0.0003 |
| 2-year | 41.7 | 31.4 | 0.006 |
| 3-year | 19.4 | 8.8 | 0.028 |
| 4-year | 13.9 | 8.8 | 0.048 |
| 5-year | 13.9 | 5.9 | 0.035 |
